# Logical Gene Ontology Annotations (GOAL): exploring gene ontology annotations with OWL

**DOI:** 10.1186/2041-1480-3-S1-S3

**Published:** 2012-04-24

**Authors:** Simon Jupp, Robert Stevens, Robert Hoehndorf

**Affiliations:** 1 European Bioinformatics Institute, Wellcome Trust Genome Campus, Cambridge, CB10 1SD, UK; 2 School of Computer Science, University of Manchester, Oxford Road, Manchester, M13 9PL, UK; 3 Department of Genetics, University of Cambridge, Downing Street, Cambridge, CB2 3EH, UK

## Abstract

**Motivation:**

Ontologies such as the Gene Ontology (GO) and their use in annotations make cross species comparisons of genes possible, along with a wide range of other analytical activities. The bio-ontologies community, in particular the Open Biomedical Ontologies (OBO) community, have provided many other ontologies and an increasingly large volume of annotations of gene products that can be exploited in query and analysis. As many annotations with different ontologies centre upon gene products, there is a possibility to explore gene products through multiple ontological perspectives at the same time. Questions could be asked that link a gene product’s function, process, cellular location, phenotype and disease. Current tools, such as AmiGO, allow exploration of genes based on their GO annotations, but not through multiple ontological perspectives. In addition, the semantics of these ontology’s representations should be able to, through automated reasoning, afford richer query opportunities of the gene product annotations than is currently possible.

**Results:**

To do this multi-perspective, richer querying of gene product annotations, we have created the Logical Gene Ontology, or GOAL ontology, in OWL that combines the Gene Ontology, Human Disease Ontology and the Mammalian Phenotype Ontology, together with classes that represent the annotations with these ontologies for mouse gene products. Each mouse gene product is represented as a class, with the appropriate relationships to the GO aspects, phenotype and disease with which it has been annotated. We then use defined classes to query these protein classes through automated reasoning, and to build a complex hierarchy of gene products. We have presented this through a Web interface that allows arbitrary queries to be constructed and the results displayed.

**Conclusion:**

This standard use of OWL affords a rich interaction with Gene Ontology, Human Disease Ontology and Mammalian Phenotype Ontology annotations for the mouse, to give a fine partitioning of the gene products in the GOAL ontology. OWL in combination with automated reasoning can be effectively used to query across ontologies to ask biologically rich questions. We have demonstrated that automated reasoning can be used to deliver practical on-line querying support for the ontology annotations available for the mouse.

**Availability:**

The GOAL Web page is to be found at http://owl.cs.manchester.ac.uk/goal.

## Background

In this paper, we explore the use of the Web Ontology Language (OWL) [1] as a practical means of delivering sophisticated querying of mouse gene product annotations such as the Gene Ontology Annotations (GOA) [2], the Mammalian Phenotype Ontology (MPO) [3] and the Human Disease Ontology (HDO) [4]. To achieve this goal, we present the Logical Gene Ontology Annotations (GOAL) tool as a practical way to query across ontologies and explore mouse gene products.

The creation of the Gene Ontology (GO) [5,6] has had a major impact on the description and communication of the major functionalities of gene products for many species. At the time of writing, GO has more than 35 000 terms for annotating gene products; it is used in more than 40 species-specific model organism databases and in cross-species databases such as UniProt and InterPro [2]. It is widely used for querying such databases, making cross-species comparisons or in data analyses, such as over-expression analysis in microarray data [7,8].

Similarly, the MPO and HDO have been used, with a lesser coverage, to indicate the associations of gene products with phenotype and disease [4,9]. The MPO is used in the Mouse Genome Informatics (MGI) database [10] to characterise the phenotypic outcome of mutagenesis experiments in mouse, and serves as a vocabulary that is being applied in the International Mouse Phenotyping Consortium [11] to semantically annotate phenotypes of knockout mice. Both the MPO and the HDO afford a rich potential for querying and analysing a gene products’ biology.

The GO and other bio-ontologies are mainly used as a controlled vocabulary to ensure genes are consistently annotated using standard terminology across many data resources; this alone offers many benefits for data integration and analysis. Bio-ontologies are, however, much more than just vocabularies; they also provide additional information about how the entities they describe are related to each other. In well-formed ontologies, these relationships have a well-defined semantics that bring added value to the ontologies [12]. For example, the hierarchical relationships allow for all kinds of a particular entity to be retrieved, as well as those with an annotation to the entity itself. These and other relationships provide support for navigation, as well as making explicit the relationship between the entities being described. It is these relationships and their associated semantics that enable communication of knowledge and the analysis of the data arising from many experiments.

For the GO, software tools such as the AmiGO browser [13], DynGO [14] or QuickGO [15]) provide interfaces to exploit the hierarchical structure of the GO and to support query expansion. For example, when searching AmiGO for receptor activity genes, the results returned also include genes involved in GPCR activity, because GPCR activity is a subclass of receptor activity. The ontologies’ hierarchical structure is also useful for data mining tasks [16]. Enrichment analysis over the GO [8] is a common technique used in the analysis of high-throughput gene expression data; sets of interesting genes can be grouped or clustered based on common GO annotations (see [17] for more GO tools).

While GO is relatively rich with query-based tools, other ontologies and their annotations are less well endowed. Only a few query tools are able to take the information in several ontologies into account when retrieving annotated data [18-20]. In particular, in the context of complex diseases and syndromes, it can be important to retrieve data based on explicit and implicit relations that hold between classes in several biomedical ontologies. For example, diseases may be related to gene products annotated with a particular molecular function and biological process, so that they produce a distinct phenotype when defective or deactivated. Relations between different kinds of entities (functions, processes, phenotypes, diseases) can be exploited both to perform expressive queries and to add background knowledge in an ontologies’ class definitions. For example, diseases can be characterized based on their phenotype, and phenotypes in turn can be characterized based on molecular functions and biological processes in an organism. These relations can be exploited to suggest causal genes for diseases, identify genes participating in the same pathways and group orthologous genes together [21,22].

Bioinformatics is, of course, replete with systems for querying data [23,24]. Cross-resource querying has long been a goal, and to achieve this goal, integration of data and associated meta-data is required. Ontologies are proposed as a means for such integration—a common *schema* and *vocabulary* across resources will ease integration, querying and subsequent data analysis [25]. TAMBIS [26] was an early attempt to use ontologies to integrate and query across multiple resources. Latterly, the adoption of ontologies across many resources has eased cross-resource query answering [5,6]. The rise of Semantic Web [27] technologies, such as the Resource description Framework (RDF) and OWL, has eased some features of integration with resources such as Bio2RDF [28] bringing together many resources in a common format and semantics. Other RDF stores have used ontologies to a greater or lesser extent [25,29]. Yet, little work has been done to bring ontologies together with the data they annotate in order to use automated reasoning to query those data using the knowledge expressed in bio-medical ontologies. Achieving this goal demands the use of complex query languages as well as highly efficient and optimised automated reasoners.

Whilst highly useful, many of the GO-orientated tools fail to exploit the full potential of the GO’s representation for reasoning and querying over gene annotations. In particular, most of the GO tools that we investigated do not facilitate rich querying that takes into account the semantics of the GO. For example, it was difficult to ask for all proteins that are located in a membrane, or part of a membrane, that are receptor proteins involved in a metabolic process. Extending the queries to include associations of gene product functional attributes, location with phenotype and disease phenomena, such as linking together proteolysis, insulin secretion, plasma membrane, increased glucose concentration and diabetes, is not yet possible. To answer such a query correctly, some form of reasoning over the ontologies is required. The ability to perform such rich queries would enable more precise and flexible exploration of the annotations with GO, MPO and HDO, as well as other ontologies used to annotate gene products.

OWL [1] and the OBO Flat File Format [30] have a strict semantics that makes it possible to use automated reasoners to help build and use knowledge captured in an ontology. In order to explore the potential of reasoning over the various gene product annotations, we need to describe the relationships between the genes and their annotation within a framework that can also exploit the semantics encoded into the ontologies. Our approach uses OWL, for which a mapping from the OBO Flat File Format has been created [30], to represent the GO, MPO and HDO based annotations and the axioms in the ontologies so that query systems can exploit both the ontology and its annotations. In doing this, we will also investigate the ability of OWL to scale to such a task and how to deliver the querying facilities on offer to users in a way that is reasonable to use without having to write complex queries.

As an ontology of biological processes, molecular functions and cellular components, GO itself does not explicitly contain classes for gene products; GO annotations are attached to gene products in databases or flat-files (See http://www.geneontology.org/GO.annotation.shtml). We can use these annotations to create explicit OWL descriptions of the relationships between gene products and their annotations. For example, the mouse gene *Taar4* [MGI:2685072] has several GO annotations including ‘integral to membrane’ [GO:0016021], ‘G-protein coupled receptor activity’ [GO:0004930] and ‘signal transduction’ [GO:0007165]. We can create an OWL class that captures the annotations using the following Manchester OWL syntax [31] (note that an axiom annotation is used to assert the evidence code for each annotation):

Class: MGI_2685072

oboInOwl: hasDefinition ”trace amine–associated receptor 4”,

rdfs: label ”Taar4”

SubClassOf:

Annotations: oboInOwl: evidenceCode ”IEA”

ro: located_in some GO: GO_0016021, (integral to membrane)

Annotations: oboInOwl: evidenceCode ”IEA”

GOAL: is_capable_of_function some GO: GO_0004930, (G–protein coupled receptor activity)

Annotations: oboInOwl: evidenceCode ”IEA”

GOAL: is_capable_of_process some GO: GO_0007165 (signal transduction)

Using the compositional approach to ontology building [32], we can create an ontology from these annotations that explicitly relates gene products to GO, HDO and MPO and then add defined classes to impose a hierarchy. To construct the GOAL ontology we take the existing GO, HDO and MPO classes and create a set of defined classes that enable us to query for gene products. For example, for the GO class G-protein coupled receptor activity we would create a new class that queries for the gene product using the following Manchester OWL syntax:

Class: GOAL: GO_0004930

rdfs: label ’G–protein coupled receptor activity gene product’

EquivalentTo:

GOAL: is_capable_of_function some GO: GO_0004930 (G–protein coupled receptor activity)

**Figure 1:**
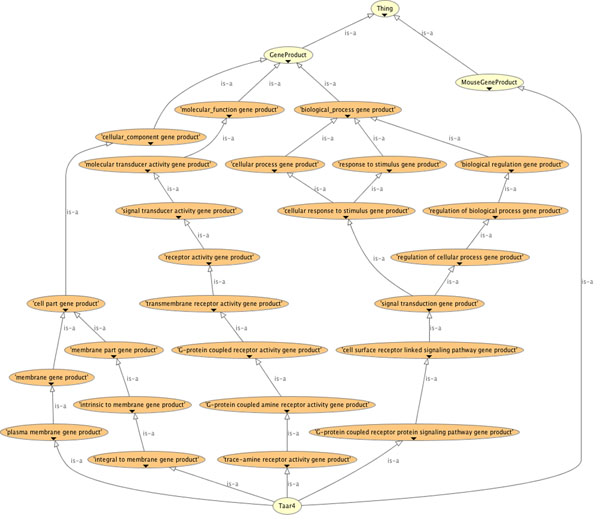
**OWLViz view of the TAAR4 superclasses.** This view shows the inferred superclass annotations for the TAAR4 gene viewed using the Protégé 4.1 OWLVIZ plugin.

This defined class will recognize any class of gene product that has these attributes, or children of these attributes, and subsume it within the hierarchy of gene products. In this standard use of OWL subsumption querying and automated reasoning, we can add more of such defined classes to build an arbitrarily complex polyhierarchy for querying and navigation of entities annotated with the GO. Figure 1 shows such an inferred polyhierarchy centered on annotations for the TAAR4 gene product.

As the use of other ontologies besides GO for annotation of gene products has spread, we can broaden this approach further. There are, however, several challenges that arise in creating a tool such as GOAL:

1. How should these annotations be represented ontologically;

2. How do we achieve appropriate performance with automated reasoners;

3. How do we enable a broad spectrum of users to access such a querying mechanism without demanding complex query syntaxes to be used;

4. How should the ontology and its queries be presented to users?

## Results

The following ontologies and annotations were downloaded on 4 October 2011 and processed as described:

• We extracted all mouse genes from the MGI database and applied our filtering, producing a total of 231 880 gene-annotation pairs.

• On conversion to OWL primitive classes this represents 17848 individual gene products.

We created the following ‘singleton’ defined classes to act as the ‘building blocks’ for GOAL queries:

• ‘molecular_function gene product’ for GO molecular function;

• ‘biological_process gene product’ for GO biological process;

• ‘cellular_location gene product’ for GO cellular location;

• ‘phenotype gene product’ for MPO classes;

• ‘disease gene product’ for HDO classes.

The numbers of these classes simply reflect the numbers of classes in each of the supporting ontologies. The total number of defined classes in the GOAL ontology is 37943.

After importing the three branches of GO, the HDO, the MPO, the GOAL ontology of named gene product classes plus the gene product annotations, the ontology contains 108226 OWL classes.

**Figure 2:**
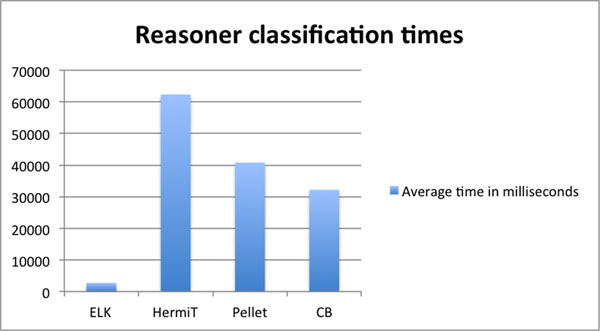
**Reasoner classification times chart**. Bar chart showing reasoner classification times in milliseconds.

Classification of the GOAL was performed on a 2.2GHz i7 Mac Book Pro requiring around 3GB of memory. Table 1 shows the performance times for each reasoner. Figure 2 shows the classification times in a chart. The ELK reasoner significantly outperforms the other three reasoners when classifying the GOAL ontology. Given that the GOAL ontology is within the OWL 2 EL profile and that ELK is specifically optimised for classifying ontologies in this profile, this result is not surprising.

**Table 1:** Reasoner classification times table

Reasoner classification times				
Reasoner	t1 (ms)	t2 (ms)	t3 (ms)	Mean time (ms)

ELK	2806	2611	2729	2715
CB	31292	31261	33988	32180
Pellet	40519	40801	41009	40776
HermiT	61461	62855	62677	62331

Reasoning times (milliseconds) for the three OWL automated reasoners used to classify the GOAL ontology.

The following queries illustrate the capabilities of the generated ontology that span the annotations from all five imported ontologies. The first query class, written in Manchester OWL syntax, returns subclasses of gene product that are annotated with immune system disease from the HDO. In addition, we know that cytokine genes are secreted by numerous cells of the immune system, so we extended the description to include genes that have a phenotype associated with abnormal cytokine secretion from the MPO. Using the GO annotations, we then reduced the result set further by filtering on genes that have the function of ion binding, participate in an inflammatory response and are located in intracellular membrane-bounded organelle s.

Class: ImmuneSystemDiseaseGeneProduct

EquivalentTo:

GOAL: GO_0006954 (’intracellular membrane–bounded organelle gene product’)

and GOAL: GO_0043167 (’inflammatory response gene product’)

and GOAL: GO_0043231 (’ion binding gene product’)

and MouseGOAL–MP: MP_0003009 (’abnormal cytokine secretion gene product’)

and MouseGOAL–HD: DOID_2914 (’immune system disease gene product’)

Using the GOAL browser, we constructed the intersection of the gene product classes and asked for all subclasses that are gene products. The following code appeared in the DL query box of the GOAL browser:

’intracellular membrane–bounded organelle gene product’

and ’inflammatory response gene product’

and ’ion binding gene product’

and ’abnormal cytokine secretion gene product’

and ’immune system disease gene product’

Running this query returned one gene, *Mefυ* (MGI:1859396). The *Mefv* gene (Mediterranean fever gene) is known to play a role in the inflammation response and in fighting infection [33].

**Figure 3:**
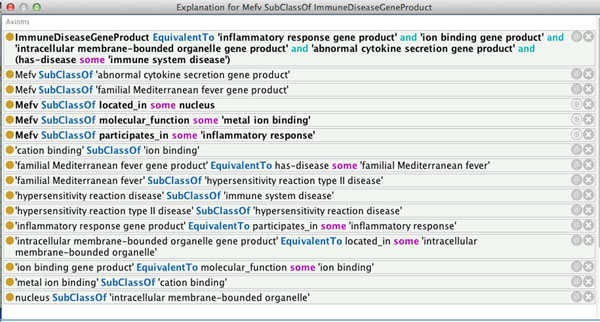
**Mefv explanations.** Explanation for the *Mefv* gene product being a subclass of the query class ImmuneSystemDiseaseGeneProduct described above. The explanation was generated using the explanation facility in Protégé 4.1.

Although this is a relatively simple query, some reasoning is required in order for it to return the correct answer. To understand what reasoning took place to answer this query, we used the explanation facility offered by Protégé. We asked the reasoner for an explanation for the *Mefv* gene’s being a subclass of our ImmuneSystemDiseaseGeneProduct class. Explanations provide the minimal set of axioms asserted in the ontology that are required for this subclass entailment to hold [34]. Figure 3 shows the explanation, in terms of the asserted axioms, for this entailment as it is shown in Protégé. We see from this explanation that the assertions on *Mefv* involved classes that are deep within the GOAL ontology’s hierarchy. The query class has restrictions on more general terms that the ones used to describe *Mefv* and it is only by inference up the hierarchy that the query can be answered. This example shows both how the reasoner can be used to answer questions about gene products, and how explanation technology can be used to provide details of how a particular query is being answered.

We can continue to construct such queries and thus begin to explore relationships between GO annotation, observed phenotypes and diseases. The ability to exploit the ontological hierarchies enables us to ask generalized queries, such as all metabolic disease genes and then start filtering down to genes with an abnormal cholesterol level phenotype, and finally down to genes that are involved in a particular biological process in the cell such as regulation of cholesterol storage. The OWL representation and reasoning make such querying and exploration trivial and there are currently no tools available that can facilitate such querying and reasoning over these sets of annotations. The results from the ELK reasoner in particular demonstrate that such interactive querying, supported through automated reasoning, is supportable for ontologies of this size that are expressed in the OWL 2 EL profile.

There are a host of example queries for users to browse via the GOAL web interface. The GOAL interface provides a mechanism to explore these annotations and develop new queries. Its current functionality demonstrates how OWL and reasoning technology can be used to deliver novel search capabilities to biomedical applications. Through GOAL, users can browse the gene product hierarchy generated for each class from the imported ontologies. Selecting a class shows the full OWL description rendered in Manchester OWL syntax. Any selected class can be added to the DL query box to create an intersecting query of named classes. Each query returns all subclasses that are gene products and links through to the gene accession page at the MGI. Users are also free to type in any arbitrary DL query using the Manchester OWL syntax.

## Conclusions

Although the queries demonstrated here are relatively simple, they serve to illustrate the potential of a pure OWL approach to querying GOA, MPO and HDO annotations. By combining the five ontologies in one place, we can make queries that connect gene products with basic functional attributes, their disease and phenotype. It is easy to imagine adding further ontologies such as cell type, gross anatomical location and so on, to provide even richer queries. We can already see queries that are not possible in other popular browsing based tools.

By keeping to the OWL 2 EL profile, we can keep the query responsiveness to reasonable limits that enable real-time automated reasoning and access to reasoning through a web interface. Moving outside this profile makes querying based on automated reasoning significantly slower, to the extent of making it currently unfeasible to use in real-time applications that rely on user interactions.

The key, on top of these features, is to provide a user interface to this querying facility. The GOAL user interface, built using GWT, on top of the OWL API means that we can offer access to the GOAL ontology and its query facilities without resorting to clumsy syntax. We have provided a straight-forward means of building reasonably complex queries over a range of ontologies. We have done this by providing what we have called “singleton” defined classes underneath our gene product classes for each of the supporting ontologies. We then do subsumption querying by making anonymous classes of conjunctions of these singleton defined classes. The classifier places these query classes in the GOAL hierarchy, providing results consisting of the primitive classes that represent the actual gene products. We see this mechanism as a reasonably straightforward means of providing sophisticated queries to users.

Using similar patterns, we can also begin to imagine more complex class descriptions that utilise additional expressivity in OWL, such as the use of complement classes to query for genes that is_capable_of_function some (not ReceptorActivity) and is_capable_of_process some SignalTransduction, which would find those genes that have a function other than receptor activity and are involved in signal transduction. (Note that the semantics of OWL implies that such genes can have a receptor activity, but must have some activity that is proven to be disjoint from any receptor activity.) We can also forsee queries that involve disjunctions—’show all gene products that participate in either Sensory perception of sound or Sensory perception of smell or both’. Such expressivity falls outside the OWL 2 EL profile that we have used and the performance of automated reasoning would be significantly worse when these constructs are used: the GOAL ontologies classifies, but response times in the user interface are currently slow enough to detract from usability (data not shown).

Adding further semantics into the ontology will afford further opportunities; adding disjointness axioms to GO may help us uncover mis-annotations and we have yet to fully exploit property characteristics such as transitivity and functionality. We can also explore ways of flexibly incorporating annotations with differing degrees of confidence through use of the GO evidence codes and programmatically generating the defined classes that form the polyhierarchy of genes.

The announcement of the GO cross-products extension to the GO [35,36] will provide rich formal definitions for some GO classes. These definitions will enable more expressive OWL queries over the GO annotations and the potential to infer more annotations on existing GOA genes [37,38]. These types of extension should also address the issue of coupling functions, processes and locations together in the GO annotations—something that will improve the computational aspects, including querying, of these annotations. While more precision in the formal representation of the ontology will generally lead to the possibility for more powerful queries, this precision and expressiveness must be balanced with the computational performance of the resulting representation; to do practical work, compromises on representation must be made when appropriate.

In this work, we have made a straight-forward use of OWL and automated reasoning to deliver a flexible way to query all aspects of GO annotations. The polyhierarchy formed also provides similarly rich navigation in a gene product orientated setting. Finally, we provide a flexible framework for exploring and manipulating GO and other valuable annotations developed by the community. By taking care with the OWL profile used and the means of issuing queries, we have shown it is possible to deliver real-time query systems for ontologies via the direct use of automated reasoners.

## Materials and methods

An overview of our method is:

1. Decide upon the GOAL representation;

2. Download GO, HDO and MPO and convert to OWL;

3. Download annotations of mouse gene products from the MGI database [10] and convert to the GOAL representation;

4. Create defined classes for each concept in the GO, MPO and HDO that links the notion of *gene product* to each of these attributes;

5. Create the GOAL ontology by importing all the bits into one master ontology;

6. Apply an automated reasoner to the GOAL ontology;

7. Offer the GOAL ontology for subsumption querying through the construction of simple subclass queries based on the pre-built defined classes.

### The GOAL representation

We take the five ontologies that describe aspects of gene products as they exist; we make no alterations to their form except to convert them into OWL and to add a ‘convenient’ root class to HDO and MPO (these two ontologies do not have a single root class, so disease gene product was created for the HDO and phenotype gene product was created for the MPO). We do this using the version of the OBO to OWL converter made available in the OWL API [39] version 3.2 downloaded from the OWL API website (http://owlapi.sourceforge.net/).

As there is no explicit representation of gene product in these ontologies, we created our own ontology to link gene products to the various aspects represented by the five ontologies. We chose the class Gene product as the top-level of our ontology as we can potentially describe both RNA and protein gene products.

Based on these considerations, the representation in OWL is fairly straight forward; for each kind of gene product, we generate an OWL class that has these gene products as instances, and we use the identifier of the kind of gene product as the class’ identifier and the gene product’s name as the class’ label. We then assert this class as a subclass of Gene product.

We use the following properties to create restrictions on Gene product classes with classes from each of the following ontologies:

Property: is_capable_of_process

Property range: GO:’biological process’

Definition: A relation between a material entity (such as a gene product) and a process. This property is asserted as a sub property of the OBO Relation Ontology capable_of in our GOAL ontology.

Property: is_capable_of_function

Property range: GO:’molecular function’

Definition: A relation between a material entity (such as a gene product) and a function. This property is asserted as a sub property of the OBO Relation Ontology capable_of in our GOAL ontology.

Property: is_located_in

Property range: GO:’cellular component’

Definition: See OBO_REL:located_in http://obofoundry.org/ro/#OBO_REL:located_in.

Property: is_associated_with_phenotype

Property range: MPO:’phenotype’

Definition: A relationship that associates members of the gene product class to at least one instance of a phenotype.

Property: is_associated_with_disease

Property range: HDO:’disease’

Definition: A relationship that associates members of the gene product class to at least one instance of a disease.

Using these properties, we generate the following Gene product class:

Class: ’Gene product’

SubClassOf: is_capable_of_function some GO: ’molecular function’,

is_located_in some GO: ’cellular component’,

is_capable_of_process some GO: ’biological process’,

is_associated_with_phenotype some MPO: ’phenotype’,

is_associated_with_disease some HDO: ’disease’

All restrictions upon the Gene product class are made with existential quantification; we ‘know’ that these relationships exist, but we do not ‘know’ that these are all the relationships that exist to these various aspects, so universal quantification cannot be used legitimately.

### Gathering ontologies and gene product annotations

The GO annotations for 25,111 mouse genes were downloaded from the MGI website (http://informatics.jax.org/) in October 2011. We filtered these genes to exclude the RIKEN cDNA genes, and also discarded annotations to root GO terms from each of the biological process, molecular function and cellular component branches.

For MPO annotations, we utilized the MGI_Geno_Disease.rpt file available from the MGI ftp site (ftp://ftp.informatics.jax.org/pub/reports/index.html). The file includes identifiers for loss-of-function mutant mouse models together with the identifier of the gene that has been targeted. We extracted the gene identifier and associated it with the observed phenotypes using the is_associated_with_phenotype relation.

From the same file (MGI_Geno_Disease.rpt), we extracted the OMIM annotations of mouse models of human disease. These annotations were manually created by curators after review of the scientific literature. The HDO provides mappings to OMIM diseases, i.e., it contains pairs of HDO classes and their equivalent OMIM diseases. We used these mappings to generate HDO-based annotations of mouse models, and associated these with the diseases in HDO using the is_associated_with_disease relation.

### Generating the OWL axioms

Instead of generating the axioms by hand, a Java program was written using the OWL API [39] to specify and instantiate the pattern for generating the class descriptions described in the introduction. For each class in the GO, MPO and HDO a new defined class was created that represented a gene product. The pattern we use is:

Class: ’?x gene product’

EquivalentTo: ’gene product’

that ?property some ?x

Where ?x is the class within GO, MPO or HDO, and ?property is substituted with the appropriate property described above. For example, for the mitochondrion class in GO we create a new class called mitochondrial gene product as follows:

Class: ’mitochondrion gene product’

EquivalentTo: ’gene product’

that is_located_in some ’mitochondrion’

Our strategy in creating such defined classes for each of the classes in GO, MPO and HDO was two-fold: It creates hierarchies of gene products over the actual classes of mouse gene products (as shown in Figure 1); This afforded a reasonably straight-forward mechanism to create more complex queries for the gene products. Our aim was to query through combining features from GO, MPO and HDO in any arbitrary combination. This will be complex if we ask users to write these subsumption queries according to the pattern for ’?x gene product’ described above. We can, however, make such queries easier by allowing simple intersecting classes to be made through the array of defined classes we generate. For instance, to ask for gene products that have a receptor activity, are participants in signal transduction and appear in the synaptic membrane, we will formulate the following query and ask the reasoner for it’s subclasses:

’signal transduction gene product’

and ’receptor activity gene product’

and ’synaptic membrane gene product’

This query is both a short form for, and logically equivalent to asking for subclasses of:

is_capable_of_process some GO: ’signal transduction’

and is_capable_of_function some GO: ’receptor activity’

and is_located_in some GO: ’syntactic membrane’

This form of querying makes it easier to develop a user interface for querying: classes are simply chosen and added to a list of classes over which to generate a defined class in the same pattern. Creating one defined class, a ‘singleton class’, for each class in GO, HDO and MPO gives sufficient building blocks for any query, whereas creating all possible combinations from the supporting ontologies is not possible and even a limited number will make for a cluttered and difficult user interface. We can still leave open the possibility of more complex queries using another OWL expressivity. These queries may utilize constructs such as disjunction. However, the disadvantages are that such queries require a more complex syntax and therefore a more complex user interface support, and raise the complexity of automated reasoning.

It is possible in this query mechanism to make queries that are biologically ‘nonsense’. The GO annotations, for instance, do not record explicitly the cellular locations in which different annotations for functions and activities take place. For example, gene products that participate in microtubule based locomotion do so only in the microtubule cellular component. Such genes may participate in other processes outside of that location, but such information is lost in the GO annotation. Therefore, it is possible to issue a query that combines function, biological process and location that recall gene products that do not hold that combination of attributes at the same time. This has long been recognised [6,40], with fixes proposed such as simple statistical co-occurance [6,40] and adding information from text-mining to incorporate this information. This is an important issue, but these approaches are only really patches for the problem. The GO, however, are releasing extensions to the GO that link between the various GO aspects [35,36,41]. For example, the occurs_in property is used to relate processes to the cellular component location at which they occur. We have used these GO extensions within GOAL and with increasing coverage of these relations, the accuracy of the enabled queries will increase.

### Classifying the GOAL ontology

All portions of the GOAL ontology have been automatically generated. In order to browse and query the ontology we needed to classify the ontology. We kept the ontology in the OWL 2 EL profile [42], as automated reasoning for the OWL 2 EL profile is tractable [43,44] and therefore enables fast querying. We explored which classifier was most rapid by using the following set of automated reasoners:

• Pellet version 2.2.0 [45];

• HermiT version 1.3.5 [46];

• CB r.12 [47];

• ELK 0.2.0 [48].

We classified the whole GOAL ontology 3 times and calculated the mean time in milliseconds for each classification. We utilised the Java ThreadMXBean library to compute thread CPU time for each classification. As the reasoners behave differently with respect to the way they load and pre-process the ontologies, we measured the time from when the reasoner is instantiated by the OWL API to the point at which the reasoner returned the answer to a query for all subclasses of OWL:Thing.

### The GOAL user interface

We created a user interface using the Google Web Toolkit (GWT) [49]. The GOAL interface has the following design principles:

• Allow elements of a simple intersecting query of named classes to be picked via browsing;

• Allow more complex queries to be issued using Manchester OWL Syntax;

• Show the subclasses that are also gene products for the generated query;

• Each gene product is shown in the results table along with its OWL description expressed in Manchester OWL syntax.

to query interactively we do not need to classify for each query. The GOAL user interface is built on top of the OWL API, so we can classify once at deploy time; then each query is constructed behind the scenes and sent to the chosen reasoner through the OWL API. The results returned are then tabulated and displayed.

## Abbreviations

GO: Gene Ontology; GOA: Gene Ontology Annotations; GOAL: Logical Gene Ontology Annotations; GWT: Google Web Toolkit; HDO: Human Disease Ontology; MGI: Mouse Genome Informatics; MPO: Mouse Phenotype Ontology; OBO: Open Biomedical Ontologies; OWL: Web Ontology Language; RDF: Resource Description Framework.

## Competing interests

The authors declare that they have no competing interests.

## Author’s contributions

SJ constructed the GOAL ontology and developed the GOAL web application. RH constructed the MPO and HDO annotation sets and ontologies. SJ, RS, and RH developed the example query classes. SJ and RS drafted the manuscript whilst all authors contributed and approved the final manuscript.
